# Looking to the past to inform the future: What eDNA from herbarium specimens can tell us about plant–animal interactions

**DOI:** 10.1002/aps3.11633

**Published:** 2025-02-05

**Authors:** Christopher Waters, Carla Hurt, Shawn Krosnick

**Affiliations:** ^1^ School of Environmental Studies Tennessee Technological University 200 W. 10th Street, Box 5152 Cookeville 38505 Tennessee USA; ^2^ Department of Biology Tennessee Technological University 1100 North Dixie Avenue Cookeville 38505 Tennessee USA

**Keywords:** conservation, eDNA, herbarium specimens, metabarcoding, plant–animal interactions

## Abstract

**Premise:**

The importance of natural history collections in modern ecological and genetic research cannot be overstated. Herbarium specimens provide historical information that can be used to investigate community ecology, phenology, and population genetics. In this study, environmental DNA (eDNA) metabarcoding and next‐generation sequencing were used to test the efficacy of detecting historical plant–animal interactions from herbarium specimen flowers.

**Methods:**

A modified eDNA isolation method and standard Illumina sequencing protocols were used. Animal eDNA was amplified using both cytochrome c oxidase subunit I (COI) and 16S primers to increase detection probability. The relationship between specimen age (0–69 years) and target taxa read depth was also investigated.

**Results:**

We generated and identified over 1.5 million sequences of animal taxa belonging to 29 clades (families or orders). These methods enabled the detection of taxa including birds, mammals, hymenopterans, lepidopterans, coleopterans, and taxa belonging to “intrafloral” communities. While herbarium specimens overall yielded less identifiable eDNA compared to fresh material, the age of the herbarium specimen negligibly affected the amount of target and/or non‐target eDNA detected in flowers.

**Discussion:**

With careful consideration of the types of data that may be obtained through sampling eDNA from herbarium specimens, these methods could prove valuable to future research on plant–animal interactions.

Natural history collections serve as invaluable scientific resources to human society. The specimens within these collections provide a diverse array of information (Webster, 2017; Besnard et al., 2018), and these data have become much more accessible in the past 25 years through massive digitization efforts (Nelson and Ellis, 2018; Hedrick et al., 2020). Beyond fundamental information such as locality, date, and details of the physical specimen itself, specimens are now regularly used to inform studies on subjects including ecology, genetics, climate change, and even human history (Phillips et al., 2023). Herbarium specimens have been increasingly used in molecular analyses (Taylor and Swann, 1994), providing sources of DNA for rare or inaccessible taxa (Albani Rocchetti et al., 2021), studies on population genetics (Wandeler et al., 2007; Rosche et al., 2022), genomic studies (McAssey et al., 2023), and exploration of biochemical pathways (Fitzgibbons et al., 2023). These specimens are particularly valuable for asking questions relating to broad‐scale ecological patterns, ranging from the introduction of invasive species (Delisle et al., 2003), changes in phenological timing (Calinger et al., 2013; Willis et al., 2017; Park et al., 2024), plant distribution modeling (Elith and Leathwick, 2007), and interactions with animals, fungi, and bacteria (Meineke et al., 2019; Bieker et al., 2020; Bianciotto et al., 2022). In many cases, the methods used in these studies were first applied successfully to living organisms and then adapted for success with herbarium specimens.

A relatively new approach emerging within the botanical community is the use of environmental DNA (eDNA) to look at biological signatures (Banerjee et al., 2022) left on plants that provide clues about interactions between the plant and its environment (e.g., herbivory, mutualisms, or pollination). The application of eDNA metabarcoding techniques (Bell et al., 2022; Lowe et al., 2022) is particularly interesting because they allow for identification of multiple species present in a single sample. Studies using eDNA from living specimens to explore plant–pollinator interactions are quickly becoming common in literature (e.g., Evans and Kitson, 2020; Gamonal Gomez et al., 2023; Kolter et al., 2023; Newton et al., 2023). In many cases, these molecular data can facilitate the detection of species interactions not observed through traditional methods. Moreover, metabarcoding facilitates the identification of taxa to the genus or species level, whereas visual censuses are often identified to the level of order or family. DNA samples collected from flowers, fruits, or leaves can signal important species interactions. For example, Walker et al. (2022) recently used eDNA metabarcoding to examine nectar feeding by bats on agave and detected visitation from the endangered Mexican long‐nosed bat. One might predict that agave herbarium specimens from this region might contain remnant eDNA from this same endangered bat, and that it could actually be more abundant in older agave specimens collected before the bat became threatened.

Using eDNA from herbarium specimens presents an exciting opportunity to examine plant–animal interactions through a historical lens. While destructive sampling of herbarium specimens is required for animal interaction eDNA analyses, there are several situations that justify the sacrifice of some plant material. Metabarcoding and real‐time quantitative PCR (qPCR) analyses using eDNA extracted from flowers may reveal novel pollinator interactions and extend historic range maps for threatened and endangered insects. These include the nine bee species and more than 40 lepidopteran species currently listed as threatened or endangered under the U.S. Endangered Species Act (U.S. Fish and Wildlife Service, 2024). Additionally, with global insect populations in decline (van der Sluijs, 2020; Wagner et al., 2021), eDNA from a combination of fresh and preserved plant material could allow researchers to examine plant–animal interactions over time without the need for direct observation. The metabarcoding of eDNA extracted from flowers of rare or inaccessible plant species could also reveal historic pollinator communities, shifts in visitor assemblages, or data from extirpated populations.

To determine if herbarium specimens can be used for metabarcoding applications such as those described above, we compared field‐collected flower samples and herbarium specimens for seven species. We consider the benefits of this approach and the effect that specimen age may have on the success of eDNA recovery. We examine the use of herbarium eDNA metabarcoding in two scenarios: the first across widespread plant taxa that are not rare or threatened, and the second within a single federally endangered plant species, *Physaria globosa* (Desv.) O'Kane & Al‐Shehbaz (U.S. Fish and Wildlife Service, 2014). The primary goals of this study are to (1) investigate whether high‐quality eDNA from floral visitors can be extracted and identified from herbarium specimens, (2) explore the relationship between the age of a herbarium specimen and the number of identifiable sequence reads from target taxa, and (3) compare floral visitor taxa diversity as determined from eDNA metabarcoding obtained from herbarium material and fresh flower samples.

## METHODS

### Specimen selection

Seven species were chosen for eDNA isolation and metabarcoding analysis. Six common species were selected based on their local abundance, different pollinator communities, and availability of specimens in Tennessee Tech University's Hollister Herbarium (HTTU; herbarium acronyms per Index Herbariorum [Thiers, 2024]) spanning multiple decades. The six common species selected were *Passiflora incarnata* L., *Lobelia cardinalis* L., *Hesperis matronalis* L., *Phlox amoena* Sims, *Hypericum frondosum* Michx., and *Blephilia subnuda* Simmers & Kral. The rare species selected was *Physaria globosa*, a federally listed species with ongoing conservation efforts in Tennessee, Kentucky, and Indiana. Permission for destructive sampling was obtained for specimens from the following herbaria: HTTU, TENN, EKY, and APSC. For all seven species, fully open and intact flowers were removed from herbarium specimens using sterilized forceps and placed into a sterile centrifuge tube. Either individual flowers or whole inflorescences were removed depending on the size and quantity of flowers present in each species. The curators of each herbarium indicated no preservatives or pesticides were directly applied to the specimens selected for sampling.

Fresh flowers were collected for the common species comparisons from the Tennessee Tech University's Native Plant Garden or along roadsides in Putnam and White counties, Tennessee. Fresh flowers for *Physaria globosa* were collected from an accessible population in Davidson County, Tennessee, with permission from the U.S. Fish and Wildlife Service and the Tennessee Department of Environment and Conservation. Flowers were placed into sterile centrifuge tubes using clean forceps and immediately frozen in the field on dry ice. Flowers from herbarium specimens and field‐collected flower samples were stored in a –80°C freezer without additional preservatives until subsequent processing for DNA isolation (Appendix 1). Two samples were collected from fresh flowers for each plant species along with the following quantities from herbarium specimens: five *Passiflora incarnata*, four *Lobelia cardinalis*, two *Hesperis matronalis*, seven *Phlox amoena*, six *Hypericum frondosum*, four *Blephilia subnuda*, and 16 *P. globosa*. All fresh floral materials for eDNA metabarcoding analyses were collected in the afternoon when it had not rained in the previous 48 hours to increase the likelihood of eDNA in the flower material.

### eDNA isolation

Total DNA isolation was performed using a Qiagen DNeasy Blood and Tissue kit (Qiagen, Hilden, Germany) with a modified protocol described here. Prior to isolation, the frozen tubes containing fresh flowers were gently tapped on the benchtop to shake loose and remove any small animals that might be frozen within the flowers. This was done to minimize bias within the samples toward detection of only those animals physically present in the flower. In a NuAire LabGard biosafety cabinet (NuAire, Plymouth, Minnesota, USA), each flower sample was placed into a 1.5‐mL screw‐cap tube with 0.5 g of 1.0‐mm‐diameter zirconia/silica beads and 180 μL of ATL buffer (Qiagen). Samples were homogenized in 2‐min intervals using a Biospec MiniBeadbeater‐16 (Biospec Products, Bartlesville, Oklahoma, USA). Homogenized samples were centrifuged at 8000 rcf for 2 min to reduce foam before the addition of 20 μL of proteinase K. The samples were thoroughly vortexed and allowed to incubate at 56°C overnight. Two hundred microliters of AL buffer (Qiagen) was then added to each tube and incubated at 56°C for an additional 10 min before adding 100% ethanol, thoroughly vortexing, and placing the samples in a –80°C freezer for at least five days to increase precipitation of eDNA. Each sample was then placed onto a DNeasy Mini spin column, and the remaining steps followed the Qiagen recommended protocol, ending with 60 μL of AE elution buffer (Qiagen). Isolation blank negative controls were made simultaneously with other samples. Autoclaved Milli‐Q purified water (MilliporeSigma, Burlington, Massachusetts, USA) was added to tubes from the same cleaned batch used during field and herbarium sample collections. These blank negative controls were then processed alongside the other samples using the same protocol.

### eDNA library preparation

Library preparation for metabarcoding followed the protocol by Bourlat et al. (2016). We used five different primer sets for eDNA amplification targeting both 16S and cytochrome c oxidase subunit I (COI) (Table 1). All primers included the standard Illumina adapter sequences, 5′‐TCGTCGGCAGCGTCAGATGTGTATAAGAGACAG for forward primers and 5′‐GTCTCGTGGGCTCGGAGATGTGTATAAGAGACAG for reverse primers. First‐round PCR was performed in duplicate with 25‐μL reactions containing 2.5 μL 10× Advantage 2 Buffer (Takara Bio, Kusatsu, Shiga, Japan), 0.2 μL Advantage 2 Polymerase mix (Takara Bio), 0.2 mM dNTPs (Promega, Madison, Wisconsin, USA), 0.5 μL of each primer (20 μM), and 3 μL of template DNA. Two PCR negative control reactions were added in each PCR reaction using molecular water in place of template DNA. A mock community was also included using genomic DNA obtained from six taxa representing mammals (*Neotoma* sp.), birds (*Archilochus* sp.), true bugs (*Oncopeltus* sp.), true flies (*Toxomerus* sp.), and two bee genera (*Hylaeus* sp. and *Andrena* sp.). The three‐step PCR thermocycler profiles included an initial denaturing of 95°C for 3 min, followed by 30 cycles of 95°C for 30 s, 48°C for 30 s, and 72°C for 30 s, and a final extension of 72°C for 10 min. PCR products were cleaned using the High‐Prep PCR magnetic bead kit (MagBio, Gaithersburg, Maryland, USA) with a 0.8:1 ratio of magnetic beads to PCR product according to the manufacturer protocol. Cleaned PCR products were pooled across loci. Second‐round index PCR was performed in 25‐µL reactions containing 2.5 μL 10× Advantage 2 Buffer, 0.2 μL Advantage *Taq* polymerase, 0.2 mM dNTPs, 0.25 μL of each index primer (Nextera XT index kit set D; Illumina, San Diego, California, USA), and 3 μL of cleaned, pooled PCR product. The thermocycler profile included an initial denaturing of 95°C for 3 min, followed by eight cycles of 95°C for 30 s, 55°C for 30 s, and 72°C for 30 s, and a final extension of 72°C for 5 min. PCR products were cleaned using the High‐Prep PCR magnetic bead kit (0.8:1.0 bead to PCR product ratio; MagBio). DNA was quantified on a BioTek HTX plate reader (BioTek Industries, Winooski, Vermont, USA) using the QuantiFluor dsDNA system (Promega) and standardized to ~5 ng/μL. Standardized PCR products were pooled across samples, and the pooled library was visualized on an Agilent 2100 Bioanalyzer (Agilent, Santa Clara, California, USA) and quantified again using the plate reader. The final library was diluted to a 2‐nM concentration and samples were sequenced in one of two separate reactions. The first reaction used a MiSeq 500 and the second a NextSeq 1000 sequencing system (Illumina) due to an upgrade of our laboratory equipment during the library preparation portion of this study.

**Table 1:** Cytochrome c oxidase subunit I (COI) and 16S primers used for eDNA amplification without Illumina adapters.

Gene	Target taxa	Forward primer	Forward sequence (5′–3′)	Reverse primer	Reverse sequence (5′–3′)	Length (bp)	Source
COI	Macroinvertebrates	BF1	ACWGGWTGRACWGTNTAYCC	BR2	TCDGGRTGNCCRAARAAYCA	316	Elbrecht et al. (2016)
COI	Metazoans	mlCOIintF‐XT	GGWACWRGWTGRACWITITAYCCYCC	jgHCO2198	TAIACYTCIGGRTGICCRAARAAYCA	313	Wangensteen et al. (2018)
COI	Birds	AWCintF4	TCCTCAATCCTGGGAGCAATCAACTT	AWCintR6	GGATTAGGATGTAGACTTCTGGGTG	278	Patel et al. (2010)
16S	Invertebrates	MOL16S_F	RRWRGACRAGAAGACCCT	MOL16S_R	ARTCCAACATCGAGGT	200	Klymus et al. (2017)
16S	Arthropods	Chiar16SF	TARTYCAACATCGRGGTC	Chiar16SR	CYGTRCDAAGGTAGCATA	348	Marquina et al. (2019)

### Bioinformatics and statistics

All sequence data were processed on the high‐performance computing cluster at Tennessee Tech University using a QIIME2 script (Bolyen et al., 2019) modified from a script originally written by Yer Lor (personal communication). Sequences were demultiplexed based on the unique primer sequences, and the primers with adapters were trimmed using cutadapt (Martin, 2011). Trimmed reads were filtered according to fragment size (retaining those >100 bp) and quality score (truncQ ≤ 2), and chimeric sequences were removed using DADA2 (Callahan et al., 2016). Forward and reverse .fastq files were merged in DADA2, and a sequence table containing counts of unique sequences was generated. Unique sequences were compared against known sequences using a BLAST+ (Camacho et al., 2009) search with >80% identity scores against three databases: the National Center for Biotechnology Information (NCBI) nucleotide (nt) database (https://www.ncbi.nlm.nih.gov/nucleotide/), COins (Magoga, 2022), and MIDORI2 (Leray et al., 2022). To ensure we did not miss any potential hits, operational taxonomic units (OTUs) that remained unidentified after BLASTing against nucleotide databases were translated and compared to the NCBI nonredundant (nr) protein database (https://www.ncbi.nlm.nih.gov/protein/) using BLASTX. The presence of any OTU in the extraction or PCR blanks were assumed to be contamination and removed from all other samples. Identified OTUs were assigned to the broad categories “target taxa” if they were macroscopic Animalia and “non‐target taxa” if the sequences were identified as bacteria, fungi, plants, nematodes, or similar. To compare the amount of eDNA in fresh flowers to herbarium specimens, the mean number of sequence reads per sample was calculated. All data manipulation was performed in the Pandas Python package (McKinney, 2010), and sequence match results were visualized using tidyverse, dplyr, ggplot2, and ggaluvial in R version 3.6.1 (Gómez‐Rubio, 2017; Brunson, 2020; R Core Team, 2021; Wickham et al., 2019, 2023). Linear regression models comparing specimen age to sequence read depth were made in R using the tidyverse, vegan, sjmisc, and ggplot2 packages (Oksanen et al., 2001; Gómez‐Rubio, 2017; Lüdecke, 2018; Wickham et al., 2019; R Core Team, 2021).

## RESULTS

### Sequencing results

The average post‐isolation DNA concentration across all samples was 95.56 ng/µL ± 59.66 ng/µL, with the lowest DNA concentration equaling 31.30 ng/µL from a fresh sample of *Hypericum frondosum*. Following a trend in other metabarcoding analyses that use both COI and 16S primers, amplification appears to have been more efficient for 16S primers (Elbrecht et al., 2016) (rarefaction curves are available in Figures S1 and S2). After removing suspected contamination, demultiplexing the Illumina sequencer output, and filtering sequences, 2,243,347 individual sequences remained. Of these sequences, 800,130 were from the three COI primer sets and 1,443,217 sequences were from the two 16S primer sets. After attempting to identify the sequences in BLAST+, 183,108 COI sequences were identified as target taxa (77% were non‐target taxa or unidentified) along with 1,443,217 16S sequences (42% were non‐target taxa or unidentified). The identified non‐target taxa sequences from both COI and 16S were a mix of Basidiomycota, Ascomycota, Oomycota, Gram‐negative bacteria, Gram‐positive bacteria, and eudicots. Some samples, from both fresh and herbarium specimens, had no sequences pass filtering. Before sequencing, every sample had some DNA according to quantification analyses, so the lack of downstream sequences in these samples is likely due to an absence of high‐quality eDNA in the starting material that failed to pass the filtering parameters.

#### General OTU identification

After identifying OTUs using BLAST+ against the nucleotide databases, many of the OTUs that passed filtering remained unidentified. Unidentified sequences were translated and compared against the NCBI nr protein database. While this did resolve a few unknown OTUs, none of the protein hits were able to be confidently identified beyond Eukaryota, Animalia, or Arthropoda. From the identified COI OTU primer sequences, the three most frequently identified taxa were members of Thripidae (29%), Formicidae (21%), and Nitidulidae (18%); similarly, for 16S primer sequences, the most frequently identified taxa by sequence read depth were members of Nitidulidae (49%), Formicidae (22%), and Thripidae (20%). For both primer sets, the majority of identified sequences were from insect taxa that spend more time in and around flowers than pollinators. However, some potential pollinator species were detected in both the fresh and herbarium specimen samples.

#### Fresh material OTU hits

Few known pollinator species were detected from fresh flower samples using COI primer sequences. *Lobelia cardinalis* and *Passiflora incarnata* had the greatest major clade OTU diversity or target taxa among the fresh flower samples for COI sequences (11 and 13, respectively). Hits from fresh COI sequences encompassed 26 major clades including several families of Hemiptera, New World blackbirds (Icteridae), tanagers (Thraupidae), hummingbirds (Trochilidae), several families of Lepidoptera, and various other terrestrial arthropod taxa. For fresh *Physaria globosa* samples, detected taxa included mites (Acari), Crambid snout moths (Crambidae), dermestid beetles (Dermestidae), sap beetles (Nitidulidae), scarab beetles (Scarabaeidae), ants (Formicidae), and thrips (Phlaeothripidae and Thripidae). No bee species or non‐midge flies were detected using COI sequences. While solitary oligolectic bees and syrphid flies are the primary pollinators of *P. globosa* (unpublished data), various moth species, sap beetles, and dermestid beetles have been observed visiting and feeding on flowers in the population where the fresh material was collected. The three bird taxa detected using the specific bird COI primers were all from *L. cardinalis* flowers (Figures S3 and S4).

Due to the higher amplification efficiency of 16S primers, OTU hits from 16S sequences encompassed 32 major clades compared to the 26 major clades detected using COI sequences. Similar to COI, *Lobelia cardinalis* and *Passiflora incarnata* had the greatest major clade OTU diversity of any of the species sampled from fresh material using 16S sequences (17 and 22, respectively). 16S OTU hits had similar taxa overall to COI sequences, but with a greater family diversity of Coleoptera, Diptera, and Hemiptera. While no bird‐specific 16S primers were included, hummingbirds, New World sparrows, and junglefowl were detected from the fresh flower material. The hummingbirds and New World sparrows were only detected from *L. cardinalis* flowers, while junglefowl DNA was found in *P. incarnata* flowers. A single bee genus, *Andrena*, was detected in fresh *P. incarnata* using 16S sequences (Figure S4).

#### Herbarium material OTU hits

**Figure 1:**
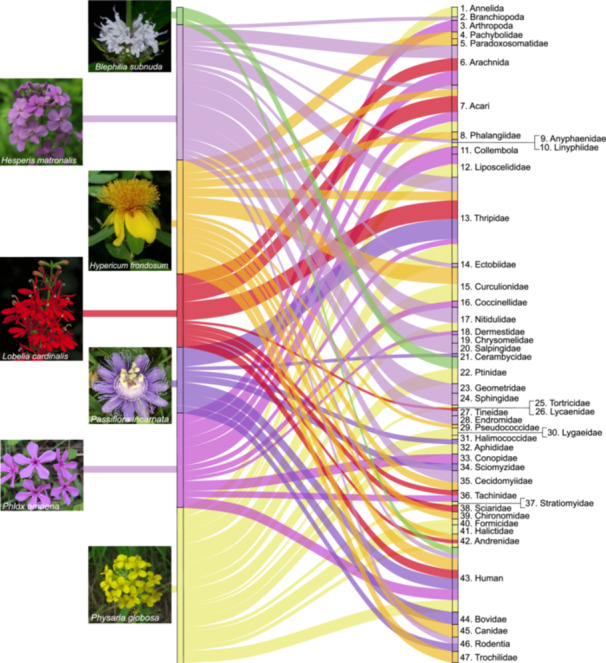
Operational taxonomic unit (OTU) matches for each plant taxon sampled from a herbarium specimen, including both cytochrome c oxidase subunit I (COI) and 16S sequences. The width of the connections corresponds to the log_10_‐transformed sequence read depth and are color coded by the flower they were extracted from. Reference images for the detected taxa are shown in Figure 2, and photograph credits for the flower images are listed in Table S1.

**Figure 2:**
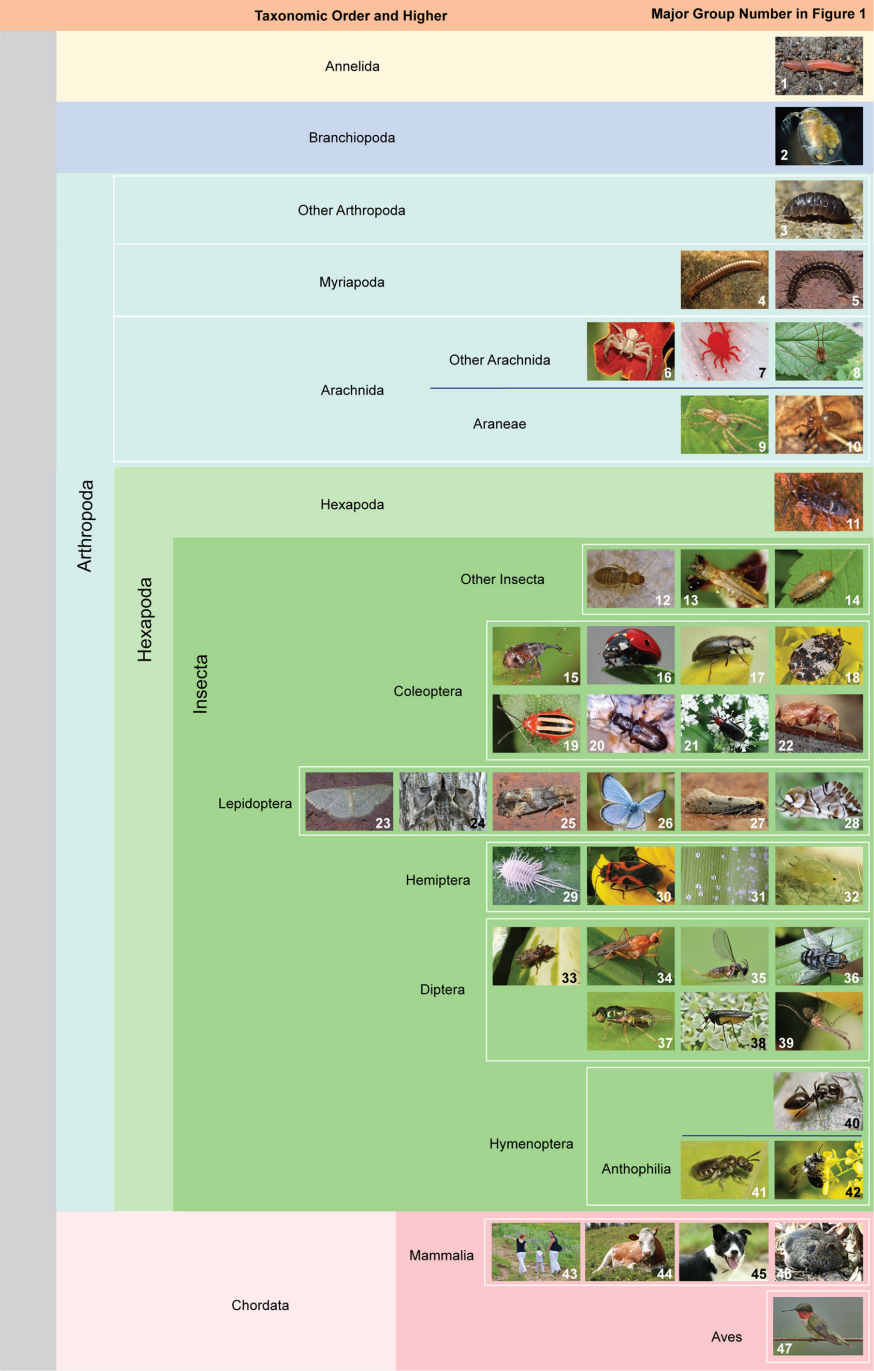
Reference photographs for the taxa detected using eDNA extracted from herbarium specimen flowers in Figure 1. Photograph credits are available in Table S1.

Using the COI primers, 29 major clades were identified from herbarium specimen OTUs mainly composed of coleopteran, dipteran, hemipteran, lepidopteran, and arachnid families. For herbarium specimens, the 16S primers led to the detection of 29 major clades that were largely similar to taxa detected with COI primers, with the addition of two bee genera. The detected bees were *Andrena* sp. from *Hypericum frondosum*, *Lobelia cardinalis*, and *Physaria globosa*, and *Lasioglossum* sp. from *P. globosa*, which is a genus of bee known to be a primary pollinator for this species (Thacker et al., 2019). The herbarium specimens also had OTU hits that included taxa that likely interacted with the specimens while in storage, including roaches (Ectobiidae), booklice (Liposcelididae), and humans. Ruby‐throated hummingbird (*Archilochus colubris*) eDNA was detected from a 51‐year‐old *H. frondosum* herbarium specimen, and the oldest herbarium specimen included in this analysis, a 69‐year‐old *P. globosa* specimen, had detectable eDNA from thrips. Most of the target taxa hits from both fresh material and herbarium specimens were from moths, beetles, and small “intrafloral” animals, which included arthropods that primarily live in or spend a significant amount of time in and around flowers feeding, mating, or seeking shelter (Figures 1 and 2).

### Effects of specimen age

**Figure 3:**
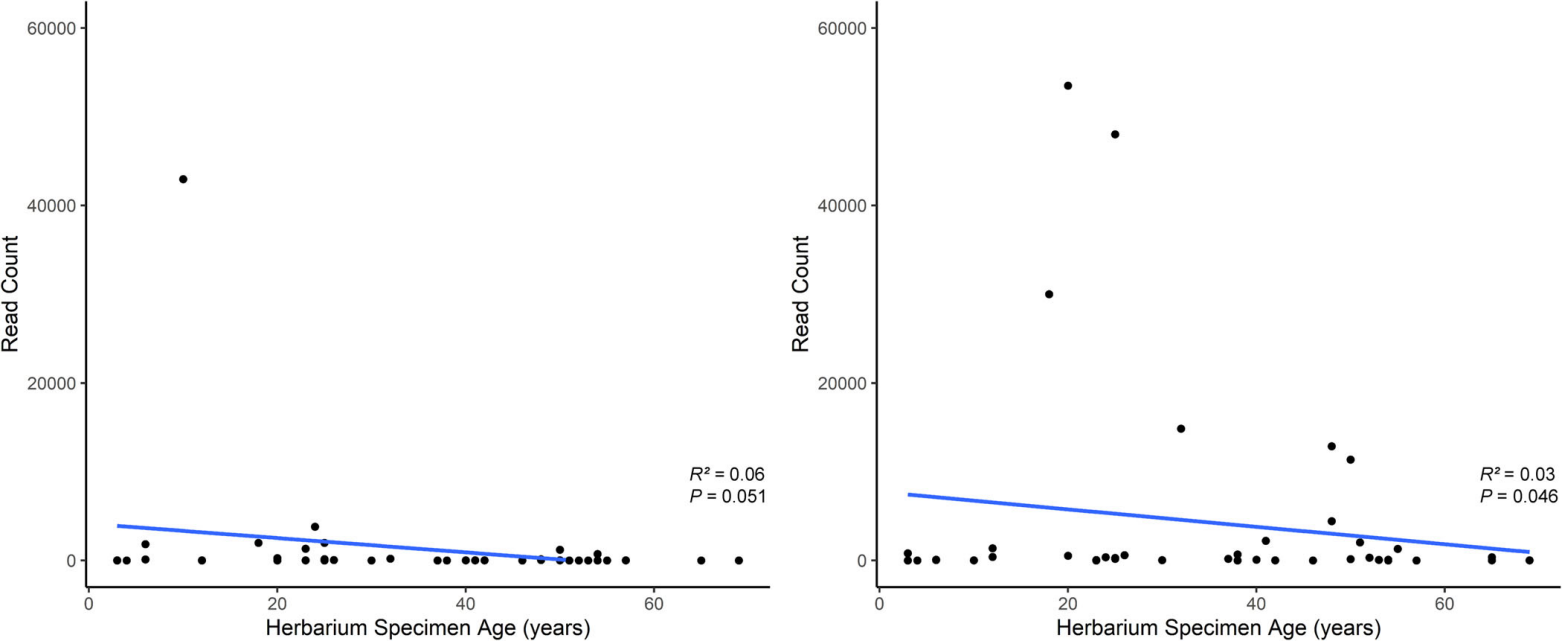
Linear regression models of specimen age for target taxa from COI (left) and 16S (right) primers, excluding fresh flower samples. Both models demonstrate a negative relationship and are negligibly significant (slope *P* values: target COI = 0.051; target 16S = 0.046). No points were removed from these data to demonstrate the variability of eDNA present in each sample.

On average, fresh flower material had more target taxa sequence reads per sample compared to the herbarium material by a factor of 10 (fresh material = 52,873 reads/sample; herbarium material = 5546 reads/sample; includes both COI and 16S reads). To investigate the relationship between the amount of detectable eDNA and herbarium specimen age, linear regression models were tested where fresh flower specimens were removed from the analysis. For COI, both the slopes of the models including all sequences (slope *P* value = 0.057) and sequences identified as target taxa (slope *P* value = 0.051) (Figure 3) were not significantly different from zero. However, while both models for 16S were still statistically significant, the slope was far less pronounced than the models that included fresh flower material (all 16S slope *P* value = 0.004; target taxa 16S slope *P* value = 0.046) (Figure 3).

## DISCUSSION

### Sequencing results

This study demonstrates that floral visitor eDNA can be successfully extracted, amplified, and identified from herbarium specimens using next‐generation sequencing and metabarcoding techniques. A combination of COI and 16S primers was used to expand the number of detected taxa. Additionally, utilizing multiple sequence databases allowed us to confirm OTU identifications and increase the confidence of the best matches. While 16S primers are more efficient than COI primers when amplified, curated reference libraries for insects are largely limited to COI sequences at the time of this study. As genomic DNA reference databases expand in the future, it is likely that unresolved OTUs will be identified at a finer resolution.

Results from the linear regression models indicated that the age of herbarium specimens did not increase the amount of non‐target OTUs or dramatically decrease the amount of usable animal eDNA in the specimens, as long as there was sufficient eDNA in the starting material (Figure 3). This remains true if all taxa that could have been introduced in a herbarium are removed from the data (e.g., humans, booklice, roaches, rodents), as these taxa accounted for only 1.88% of herbarium specimen target taxa sequence reads. However, there appeared to be under‐representation and over‐representation of some taxa in the metabarcoding results, including an over‐representation of intrafloral communities and taxa that have a greater propensity to shed biological material, while taxa that shed less or have chemical inhibitors are under‐represented in the results.

#### Over‐represented and under‐represented taxa

Some taxa appeared to be over‐represented in the metabarcoding results when compared to what was expected based on known pollinator assemblages of *Hypericum* sp. (Robertson, 1928; Boyle and Menges, 2001), *Hesperis matronalis* (Francis et al., 2009), *Passiflora incarnata* (May and Spears Jr., 1988), *Lobelia cardinalis* (Robertson, 1928; Johnston, 1991), *Phlox* sp. (Robertson, 1928; Landis et al., 2018), *Blephelia* sp. (Robertson, 1928), and *Physaria globosa* (Thacker et al., 2019; unpublished data). These results can be considered in two ways: first, over‐represented taxa may belong to intrafloral communities. Examples of insects found within these communities include thrips (Thysanoptera) and small beetles (Nitidulidae) (Nadel and Peña, 1994; Reitz, 2009). The increased amount of time these insects spend in and around the flowers will increase the likelihood of them leaving behind eDNA on the flowers. Additionally, due to the minute size of some intrafloral species, especially thrips and mites, it is likely some entire organisms were present in the flowers during DNA extraction.

Alternatively, taxa may be over‐represented in the metabarcoding results because they are physically more likely to shed material containing DNA on or within the flowers during visitation. Vertebrates, soft‐bodied organisms, and other animals with loose hairs or scales are more likely to shed skin cells, hairs, and feathers while interacting with flowers compared to other insects. This could explain why lepidopterans (Geometridae, Sphingidae, Tortricidae, Lycaenidae, Tineidae, and Endromidae) were represented more in our metabarcoding results compared to bees and flower flies. Lepidopterans may shed wing scales and hairs while visiting flowers, which would increase the likelihood of them leaving behind detectable eDNA. Similar phenomena have been documented (Tréguier et al., 2014; Adams et al., 2019), as described in the “shedding hypothesis,” which explains how organisms may leave different quantities of eDNA on environmental substrates depending on their body plan, life cycle, and behavior.

The primary taxa that appear to be under‐represented in the metabarcoding results are bees and flower flies (Syrphidae). While generalist bees and flower flies are documented to visit and pollinate most of the plant genera included in this study (May and Spears Jr., 1988; Boyle and Menges, 2001; Francis et al., 2009; Thacker et al., 2019; Murray et al., 2024), only two genera of bees (*Andrena* sp. and *Lasioglossum* sp.) and no syrphid flies were detected. The most notable absence from the data are carpenter bees (*Xylocopa* sp.), which are the primary pollinators of *Passiflora incarnata* and commonly rest on *Passiflora* flowers during the day (Hardin et al., 1972; May and Spears Jr., 1988). No *Xylocopa* DNA was detected from any flowers, fresh or pressed. While primer bias could play a role in these missing flies as they did not appear in the mock community sample, the two bee taxa included in the mock community were detected.

Bees and syrphid flies may not shed enough material to leave behind significant amounts of eDNA due to their grooming behaviors. Both bees and flies are known to frequently groom themselves when resting, which may decrease the chance they leave behind DNA‐containing material on flowers they visit (Thomson and Plowright, 1980; Wellington and Fitzpatrick, 1981; Fitzpatrick and Wellington, 1983; Thomson, 1986; Ellis and Johnson, 2012). One common bee taxon that did not appear in the metabarcoding results, *Bombus* sp., has been documented to exhibit grooming behaviors in flight between floral visits (Thomson, 1986). There is also evidence that bee saliva contains enzymes and peptides capable of degrading DNA including defensins, hymanoptaecin, apidaecin, amylase, invertase, and acid phosphatase (Casteels et al., 1993; Cruz‐Landim and Reginato, 2001; Li et al., 2006; Ilyasov et al., 2012; Danihlík et al., 2015; Shinkhede and Tembhare, 2016). In particular, some defensin antimicrobial peptides have cytotoxic effects and are known to cause damage to DNA (Amerikova et al., 2019). More research regarding the effects these salivary secretions have on eDNA is needed to understand if they are playing a role in limiting detection of bees in flowers.

### Effects of specimen age

Based on our results, we concluded that herbarium specimens will have less usable eDNA from target taxa compared to fresh flowers, but the herbarium specimens are not likely to lose a significant amount of usable eDNA over time. Older herbarium specimens tended to have less eDNA than more recent specimens and fresh material, but a relationship between specimen age and detectable eDNA from herbarium specimens was negligible (Figure 3). While it may be possible that older herbarium specimens could have more hits from non‐target taxa (i.e., fungi and bacteria) due to exposure during handling and storage, our results suggest this may not be a significant concern as all linear models resulted in a negative relationship.

When collecting fresh flowers for this study, methods were used to maximize the chances of obtaining eDNA. Flowers were only collected during peak flowering season for each species on afternoons when it had not rained in the previous 48 hours. These conditions that could affect the presence and quality of eDNA on flowers (e.g., time of day, recent precipitation, and temperature) are not typically included on herbarium labels; therefore, it is not possible to predict whether there will be any eDNA in a herbarium specimen when it is selected for eDNA metabarcoding analysis. The largest limitation for eDNA detection of pollinators from flowers is that some flowers may not have been visited by any animals prior to collection. To increase the probability of obtaining eDNA from herbarium specimens, several fully open flowers from various stems of different ages on the same specimen should be used if it is practical to do so. Additionally, any future research using herbarium specimens for pollinator eDNA metabarcoding analysis should responsibly utilize as many specimens as possible, because many of the samples may not have any detectable eDNA. However, researchers using similar methods should take care to balance the destructive sampling of herbarium specimens with realistic expectations of what usable data could be obtained.

It is currently very difficult to infer abundance from eDNA metabarcoding data outside of highly controlled experimental conditions or closed aquatic systems (Lacoursière‐Roussel et al., 2016; Yates et al., 2019; Spear et al., 2021). However, recent advances in bioinformatics could make inferring abundance from eDNA a possibility in the near future (Gold et al., 2022; Ruiz‐Ramos et al., 2023; Kelly et al., 2024). Identifying historic pollinator and intrafloral assemblages from preserved plant material, even at the genus or family level, could provide valuable data for conservation efforts. It could also provide insights into the evolution of pollinator guilds and shifts in plant–animal interaction over time, and may be a useful tool to answer many questions in the future. Additionally, the methods outlined in this study add to the many use cases of preserved specimens in herbaria and other natural history collections.

## AUTHOR CONTRIBUTIONS

The study was conceived by S.K. and implemented by C.W. Sample collection was performed by C.W. and S.K. Lab work was completed by C.W., S.K., and C.H. Bioinformatic and statistical analyses were conducted by C.W. Figures were prepared by C.W. and S.K. All authors contributed to the writing and revision of the manuscript and approved the final version.

## Supporting information


**Figure S1.** Cytochrome c oxidase subunit I (COI) sequencing rarefaction curves.
**Figure S2.** 16S sequencing rarefaction curves.
**Figure S3.** Fresh flower COI operational taxonomic unit (OTU) match alluvial plot (log_10_ transformed).
**Figure S4.** Fresh flower 16S OTU match alluvial plot (log_10_ transformed).

**Table S1.** Photograph credit information for the images used in Figures 1 and 2.

## Data Availability

Tables, figures, supplementary materials, .fasta sequence files, OTU tables, and the code templates used in this study are deposited in the Figshare project “2024 Herbarium eDNA” (https://figshare.com/projects/2024_Herbarium_eDNA/218248).

## Appendix 1 Herbarium specimen voucher information for specimens that were destructively sampled, including the age, original collection location, and material removed from each specimen.

**Table:**

Sample ID	Species	Specimen ID	Date collected	County	State	Sample description	Specimen age (years)
Blsu1	*Blephilia subnuda*	HTTU031528	17 May 1976	Madison	AL	1 inflorescence	46
Blsu2	*Blephilia subnuda*	HTTU034852	19 May 1976	Jackson	AL	1 inflorescence	46
Blsu3	*Blephilia subnuda*	HTTU035694	15 May 2019	Putnam	TN	1 inflorescence	3
Blsu4	*Blephilia subnuda*	HTTU035695	15 May 2019	Putnam	TN	1 inflorescence	3
Blsu 1F	*Blephilia subnuda*	Fresh material	29 May 2022	Putnam	TN	1 inflorescence	0
Blsu 2F	*Blephilia subnuda*	Fresh material	29 May 2022	Putnam	TN	1 inflorescence	0
Hema1	*Hesperis frondosum*	HTTU036203	26 May 2016	Rockingham	VA	4 flowers	6
Hema2	*Hesperis frondosum*	HTTU005827	20 May 1972	Smith	TN	5 flowers	50
Hema 1P	*Hesperis frondosum*	Fresh material	29 May 2022	Putnam	TN	10 flowers	0
Hema 2P	*Hesperis frondosum*	Fresh material	29 May 2022	Putnam	TN	10 flowers	0
Hyfr1	*Hypericum frondosum*	HTTU028332	5 July 1999	Jackson	TN	1 flower	23
Hyfr2	*Hypericum frondosum*	HTTU033354	7 June 2018	DeKalb	TN	1 flower	4
Hyfr3	*Hypericum frondosum*	HTTU017342	19 June 1984	Wilson	TN	1 flower	38
Hyfr4	*Hypericum frondosum*	HTTU017343	19 June 1984	Wilson	TN	1 flower	38
Hyfr5	*Hypericum frondosum*	HTTU017346	21 June 1974	DeKalb	TN	1 flower	48
Hyfr6	*Hypericum frondosum*	HTTU034799	18 June 1971	Putnam	TN	1 flower	51
Hyfr 1F	*Hypericum frondosum*	Fresh material	29 May 2022	Putnam	TN	1 flower	0
Hyfr 2F	*Hypericum frondosum*	Fresh material	29 May 2022	Putnam	TN	1 flower	0
Loca1	*Lobelia cardinalis*	HTTU024196	13 September 2012	Columbia	AR	2 flowers	10
Loca2	*Lobelia cardinalis*	HTTU024354	11 September 1999	DeKalb	TN	2 flowers	23
Loca3	*Lobelia cardinalis*	HTTU013733	16 August 1972	Putnam	TN	2 flowers	50
Loca4	*Lobelia cardinalis*	HTTU013708	31 July 1965	Ballard	KY	2 flowers	57
Loca1FB	*Lobelia cardinalis*	Fresh material	14 September 2022	Putnam	TN	3 flowers	0
Loca2FB	*Lobelia cardinalis*	Fresh material	14 September 2022	Putnam	TN	1 flower	0
Pain1	*Passiflora incarnata*	HTTU028766	9 June 2016	White	TN	1 flower	6
Pain2	*Passiflora incarnata*	HTTU027851	3 September 2004	Putnam	TN	1 flower	18
Pain3	*Passiflora incarnata*	HTTU018664	5 September 1997	Jackson	TN	1 flower	25
Pain4	*Passiflora incarnata*	HTTU018665	31 July 1970	DeKalb	TN	1 flower	52
Pain5	*Passiflora incarnata*	HTTU011358	16 July 1967	Abberville	SC	1 flower	55
Pain1FB	*Passiflora incarnata*	Fresh material	14 September 2022	Putnam	TN	2 flowers	0
Pain2FB	*Passiflora incarnata*	Fresh material	14 September 2022	Putnam	TN	2 flowers	0
Pham1	*Phlox amoena*	HTTU019118	19 April 1998	Scott	TN	2 flowers	24
Pham2	*Phlox amoena*	HTTU034105	15 May 1997	Scott	TN	2 flowers	25
Pham3	*Phlox amoena*	HTTU034107	15 May 1997	Scott	TN	2 flowers	25
Pham4	*Phlox amoena*	HTTU012094	6 May 1968	Cherokee	NC	2 flowers	54
Pham5	*Phlox amoena*	HTTU012115	7 May 1968	Cumberland	TN	2 flowers	54
Pham6	*Phlox amoena*	HTTU012088	18 May 1968	Fentress	TN	2 flowers	54
Pham7	*Phlox amoena*	HTTU012119	25 April 1969	White	TN	2 flowers	53
Pham 1P	*Phlox amoena*	Fresh material	29 May 2022	White	TN	10 flowers	0
Pham 2P	*Phlox amoena*	Fresh material	29 May 2022	White	TN	10 flowers	0
EKU1	*Physaria globosa*	EKY31234100343043	17 May 1990	Franklin	KY	8 flowers	32
EKU2	*Physaria globosa*	EKY31234100343084	May 1980	Franklin	KY	8 flowers	42
EKU3	*Physaria globosa*	EKY31234100732823	30 March 1992	Trousdale	TN	8 flowers	30
EKU4	*Physaria globosa*	EKY31234100343076	13 May 1957	Fayette	KY	8 flowers	65
EKU5	*Physaria globosa*	EKY31234100343019	13 May 1957	Fayette	KY	8 flowers	65
TENN1	*Physaria globosa*	TENN0107491	6 May 1982	Davidson	TN	8 flowers	40
TENN2	*Physaria globosa*	TENN0244327	15 April 2002	Trousdale	TN	8 flowers	20
TENN3	*Physaria globosa*	TENN0107494	26 April 1953	Davidson	TN	8 flowers	69
TENN4	*Physaria globosa*	TENN0107499	7 May 1968	Montgomery	TN	8 flowers	54
APSC1	*Physaria globosa*	APSC0083443	5 May 1981	Franklin	KY	8 flowers	41
APSC2	*Physaria globosa*	APSC0007172	17 April 2010	Montgomery	TN	8 flowers	12
APSC3	*Physaria globosa*	APSC0040148	29 April 2010	Montgomery	TN	8 flowers	12
APSC4	*Physaria globosa*	APSC0091722	6 May 1985	Franklin	KY	8 flowers	37
APSC5	*Physaria globosa*	APSC0096941	15 April 2002	Trousdale	TN	8 flowers	20
APSC6	*Physaria globosa*	APSC0045306	24 April 1996	Davidson	TN	8 flowers	26
APSC7	*Physaria globosa*	APSC0053560	16 April 1974	Davidson	TN	8 flowers	48
