# Supplementary material for: Looking to the past to inform the future: What eDNA from herbarium specimens can tell us about plant–animal interactions
Source: Appl Plant Sci. 2025 Feb 5;13(2):e11633. doi: 10.1002/aps3.11633 (PMC12038733; doi:10.1002/aps3.11633)
Supplement: Supplementary file 1 — Figure S1. Cytochrome c oxidase subunit I (COI) sequencing rarefaction curves. Figure S2. 16S sequencing rarefaction curves. Figure S3. Fresh flower COI operational taxonomic unit (OTU) match alluvial plot (log10 transformed). Figure S4. Fresh flower 16S OTU match alluvial plot (log10 transformed). [file APS3-13-e11633-s002.docx]

Supplemental figures for “Looking to the past to inform the future: What eDNA from herbarium specimens can tell us about plant–animal interactions”


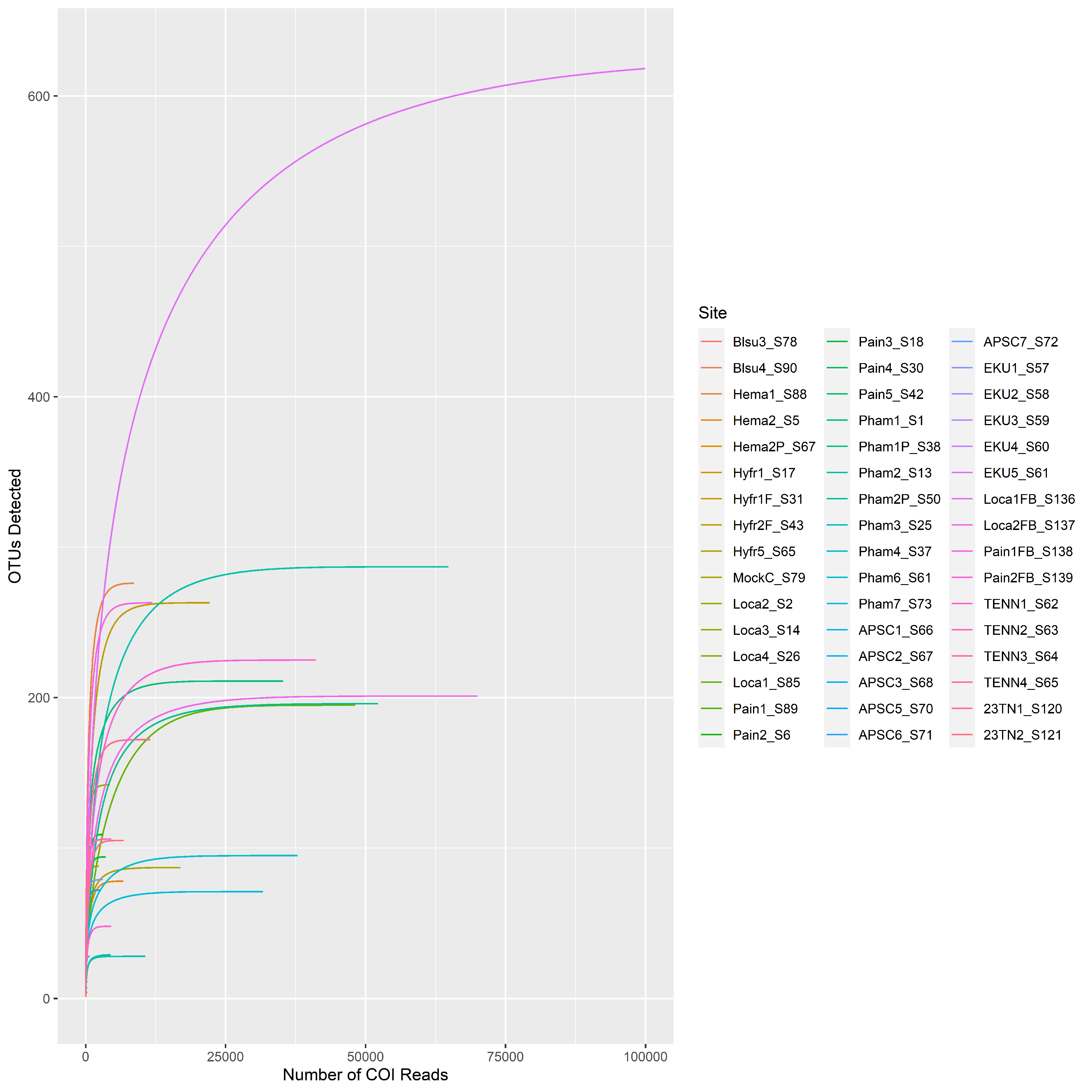


Figure S1. Cytochrome c oxidase subunit I (COI) sequencing rarefaction curves.


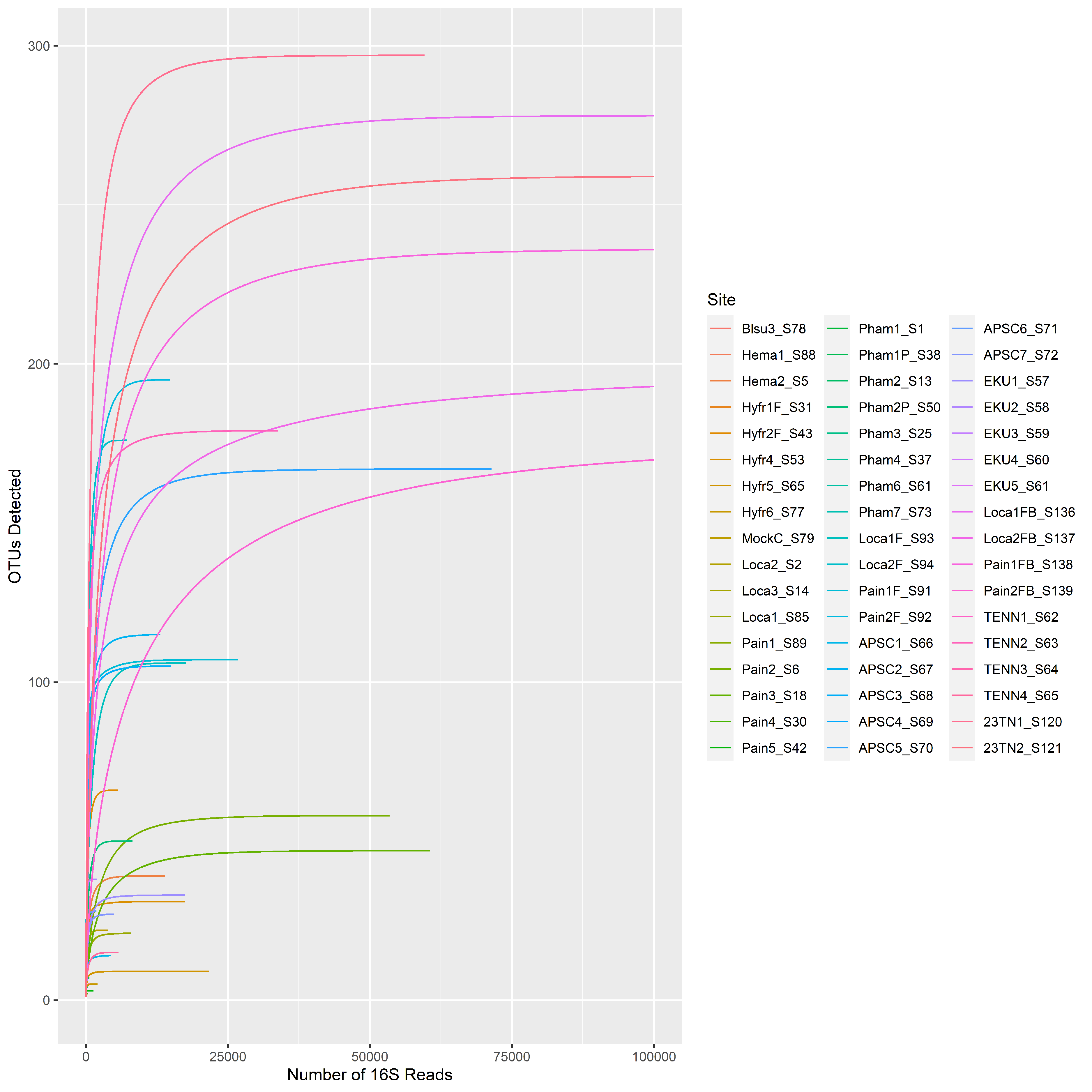


Figure S2. 16S sequencing rarefaction curves.


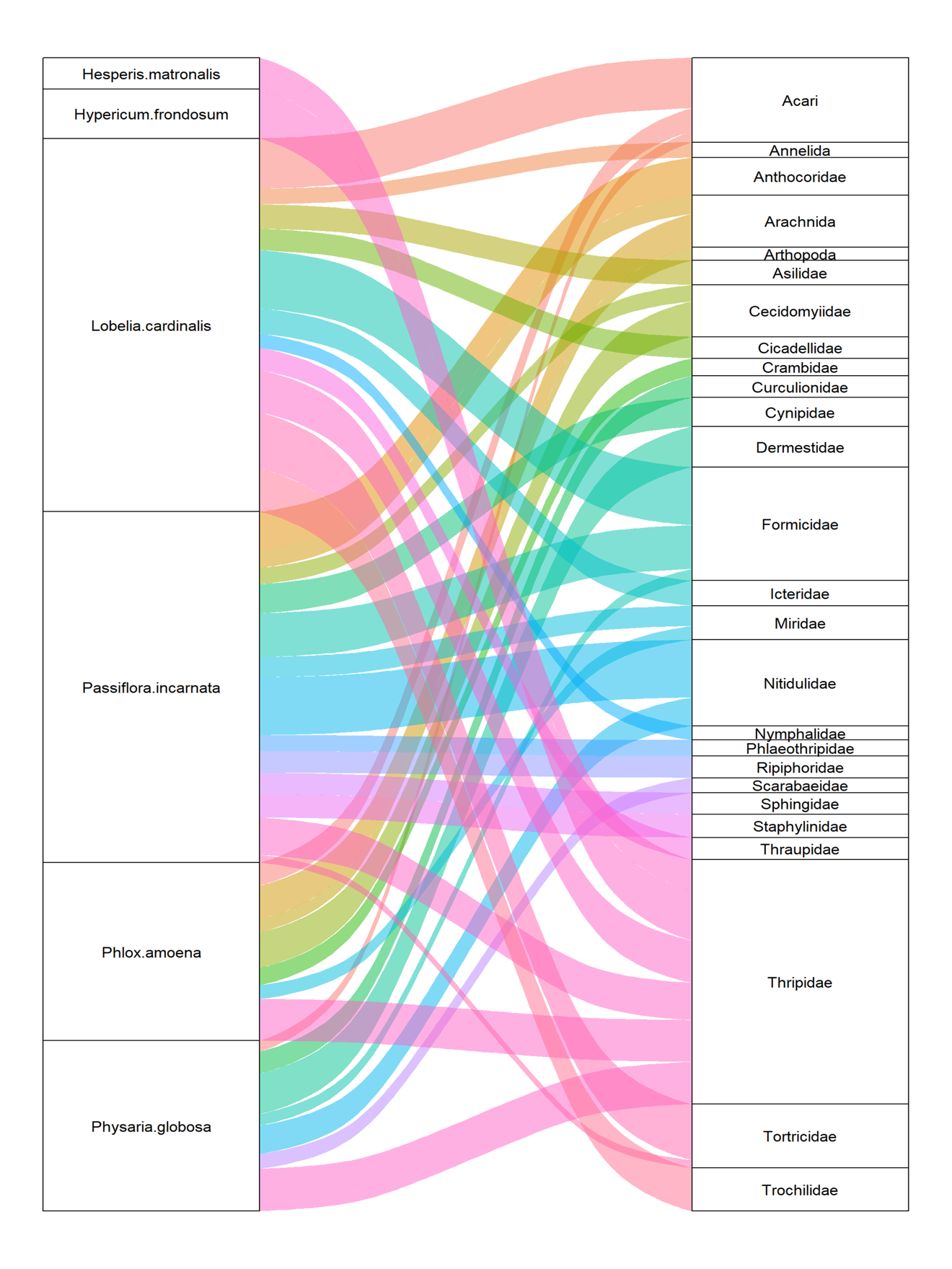


Figure S3. Fresh flower COI operational taxonomic unit (OTU) match alluvial plot (log_10_ transformed).


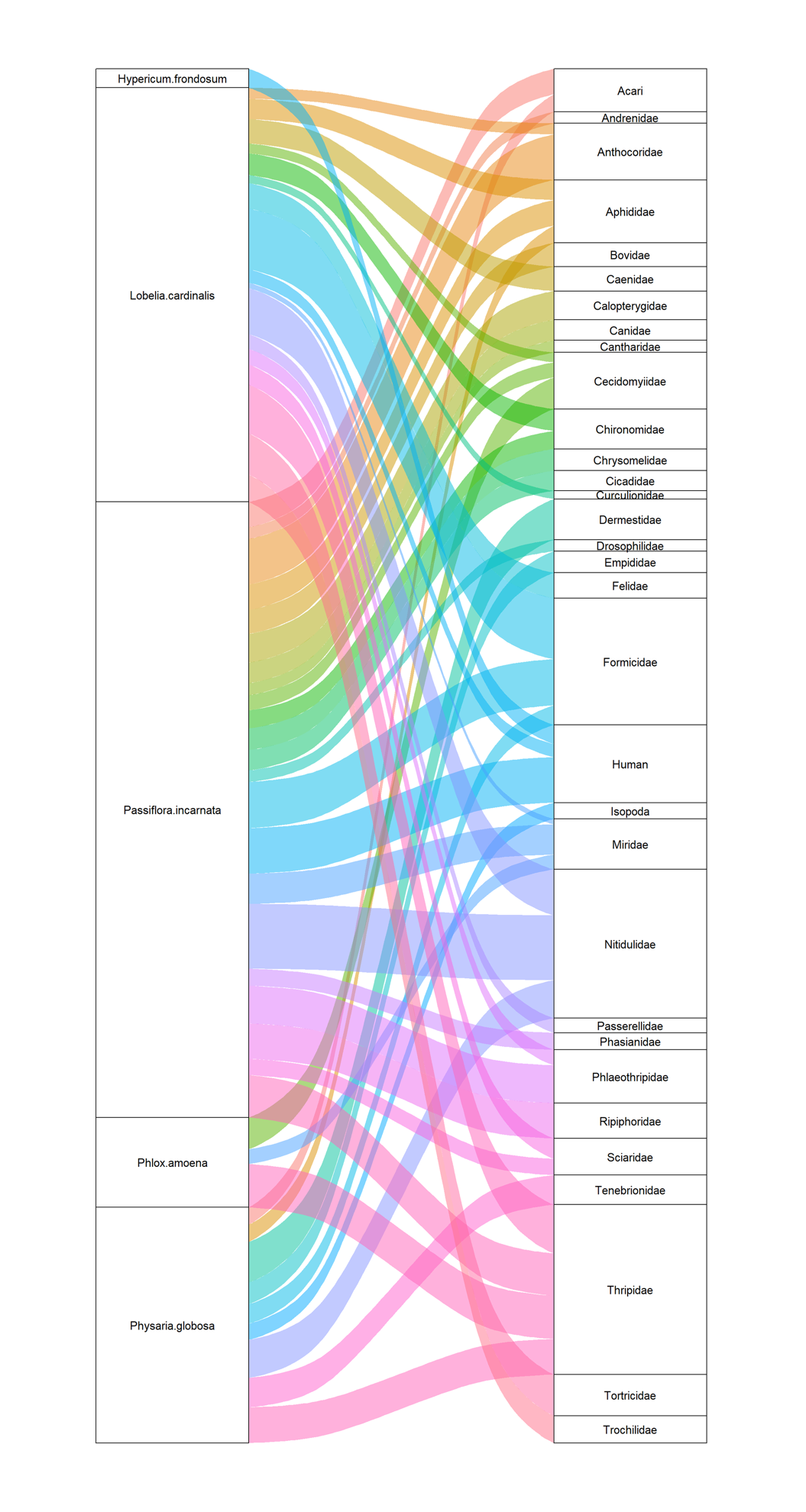


Figure S4. Fresh flower 16S OTU match alluvial plot (log_10_ transformed).
